# Zelsuvmi for Molluscum Contagiosum: Clinical Evidence, Mechanisms, and Therapeutic Significance

**DOI:** 10.1097/MS9.0000000000004333

**Published:** 2025-11-17

**Authors:** Ajeet Singh, Laiba Shakeel, Tayyaba Ashraf, Priya Goyal, Amogh Verma

**Affiliations:** a Department of Internal Medicine, Dow University of Health Sciences, Karachi, Pakistan; b Department of Internal Medicine, Dayanand Medical College and Hospital, Ludhiana, Punjab, India; c Department of Internal Medicine, Rama Medical College Hospital and Research Centre, Pilkhuwa, Hapur, Uttar Pradesh, India; d Medicos in Research, Nautanwa, Maharajganj, Uttar Pradesh, India

**Keywords:** cryotherapy, curettage, erythema, molluscum contagiosum, zelsuvmi

## Abstract

Molluscum contagiosum (MC) is a highly contagious viral dermatologic disease that predominantly affects children, immunocompromised individuals, and sexually active young adults. This skin condition, characterized by raised, flesh-colored papules with central umbilication, is caused by the MC virus (MCV), a member of the poxviridae family. While typically self-limiting, MC can lead to cosmetic concerns, discomfort, and psychosocial impacts. Traditional treatments, such as cryotherapy and curettage, are often painful and may not align with patient-centered care, highlighting the need for better therapeutic options. The recent FDA approval of Zelsuvmi, a topical gel containing 10.3% berdazimer, marks a significant milestone in MC management. Berdazimer, a nitric oxide-releasing agent, inhibits viral replication and exhibits immunomodulatory effects, offering dual-action efficacy in treating MC. Clinical trials, including the pivotal B-SIMPLE4 trial, demonstrated that berdazimer significantly reduces lesion counts, with a favorable safety profile and minimal systemic absorption. In trials, Zelsuvmi showed superior clearance rates compared to vehicle treatments, with notable efficacy even in patients with atopic dermatitis. Common side effects were mild and included localized skin reactions, such as erythema and application site pain. The introduction of Zelsuvmi provides healthcare professionals with a potent, well-tolerated option for managing MC. This advancement addresses a critical gap in MC care, offering improved outcomes and enhanced quality of life for patients.

## Introduction

Molluscum contagiosum (MC) is a highly contagious, benign neoplastic, infectious dermatologic disease^[1]^ that represents around 1% of all skin conditions worldwide, with a noticeable increase in occurrence^[2]^. It is frequently observed in immunocompromised individuals, children, and young adults who are sexually active. MC belongs to the poxviridae family, and the ratio of active infections in children to adults is 9:1. MC virus (MCV) has four subtypes: MCV-1, MCV-2, MCV-3, and MCV-4. Overall, MCV-1 is most typically observed (75–96%), and pediatric patients are particularly frequently affected. Human immunodeficiency virus (HIV) patients usually have MCV-2. Infections with MCV-3 and MCV-4 are very uncommon and are more prevalent in Asia and Australia^[1]^.

**HIGHLIGHTS**

- Zelsuvmi, containing 10.3% berdazimer, is the first FDA-approved treatment targeting molluscum contagiosum (MC) by inhibiting viral replication and boosting immune response.
- In the B-SIMPLE4 trial, 32.4% of patients achieved complete lesion resolution at week 12, significantly outperforming the vehicle group (19.7%).
- Zelsuvmi not only reduces lesions but also improves quality of life and emotional well-being, addressing both physical and psychosocial impacts of MC.
- Berdazimer gel has minimal systemic exposure, with mostly mild, localized adverse effects, making it a safe option for children and vulnerable populations.

The analysis of the MCV genome reveals around 70 unique open reading frames not found in other poxviruses, some of which contribute to immune evasion and skin persistence. These include MC159, a death effector domain-containing apoptosis inhibitor that prevents cell death induced by immune responses; MC66, a selenocysteine-containing glutathione peroxidase that protects against oxidative stress; MC148, a chemokine antagonist that disrupts leukocyte migration; and MC54, an IL-18 binding protein that inhibits immune activation. Additionally, MC80, a homolog of MHC class I, binds β2-microglobulin, although its exact function remains unclear. These viral proteins help MCV evade both innate and adaptive immune responses, promoting viral survival and spread in the skin^[3]^. Given the absence of a definitive curative therapy for MC, the prolonged course of the disease often adds to psychological stress, making it crucial to consider both physical and emotional impacts. Approximately 10% of children with MC experience significant functional and emotional impairment, often worsened by parental anxiety, fear of contagion, and prolonged or recurrent lesions, leading to school or activity exclusion^[4]^. In adults, subsequent studies report self-consciousness and embarrassment as key psychosocial impacts, mainly due to lesion visibility and contagiousness. This burden underscores the unmet need for effective, safe, and accessible treatments. Recently, there has been heightened interest in advancing MC management and mitigating its symptoms; therefore, we herein present a recent discovery.

This review focuses on the recently approved FDA drug, Zelsuvmi, which is a once-daily topical therapy for treating MC and provides a comprehensive understanding of its mechanism of action and its compelling potential in effectively addressing the dermatological condition. This manuscript complies with the TITAN Guidelines 2025^[5]^.

## Disease manifestations

MC manifests as small, raised, flesh-colored bumps on the skin, commonly found in the genital area, abdomen, arms, and legs. The hallmark of MC is the appearance of skin-colored, smooth papules with a central umbilication. In children, MC lesions are infrequent on the palms and soles and often occur on the trunk, face, and limbs. MC lesions are typically found on the genitalia, belly, and thighs in immunocompromised people; however, they can also be more widespread.^[1]^

## Risk factors

MC is influenced by factors such as exposure in infancy (e.g., daycare), higher incidence in children with atopic dermatitis, and increased immunity in adults from prior exposure. Adults generally have higher seroprevalence of anti-MCV antibodies (6–30%), which may explain the lower incidence of MC in adults^[1]^. However, recovery does not prevent recurrence, and MCV spreads through direct contact, fomites, and autoinoculation. It is more common in individuals with compromised skin barriers, like those with atopic dermatitis^[1]^. While typically self-limiting, MC can cause discomfort and cosmetic concerns^[6]^. Treatment focuses on lesion removal and preventing spread.

## Diagnosis and clinical course

MCV infects only keratinocytes and remains confined to the epidermis, preventing systemic spread or latent infection. It suppresses the innate immune response through several mechanisms, as mentioned earlier, prolonging skin lesions^[3,6]^. MCV upregulates epidermal growth factor receptors (EGFRs) to enhance cell division. During infection, virions are seen in the stratum basale, and as keratinocytes migrate to the stratum corneum, the virus replicates, forming eosinophilic inclusion bodies called Henderson-Paterson bodies or molluscum bodies in the cytoplasm. The virus is then shed from the stratum corneum, facilitating transmission. The absence of basement membrane invasion likely explains the low recurrence rate^[1]^.

## Previous treatment options

MC, a viral skin infection marked by raised, pearl-like lesions, is often left untreated due to its benign and self-limiting nature. However, this lack of intervention raises important research questions, particularly about the psychosocial impacts of the condition and the need for more patient-centered treatment options. Existing therapies (summarized in Tables 1 and 2), such as cryotherapy and curettage, are often associated with pain and inconvenience, leading many patients to discontinue treatment. This limitation underscores the need for innovative treatments that prioritize both efficacy and patient comfort.

**Table 1:** 

Treatment	Administration	Key points^[7]^
Cantharidin	By clinician	Applied topically; suitable for adults and children aged 2 years and older.
Cimetidine	Self-administered	Oral prescription medication; beneficial for patients with atopic dermatitis or widespread lesions.
Cryotherapy	By clinician	Device-based treatment; can be painful, making it less ideal for young children or patients with extensive lesions.
Curettage	By clinician	Involves scraping; effective for older children, teenagers, and adults.
Imiquimod cream	Self-administered	Topical prescription; not recommended for younger children.
Pulsed dye laser	By clinician	Device-based; effective for patients with numerous or treatment-resistant lesions, such as those with AIDS; may cause temporary skin pigmentation changes; high cost.
Salicylic acid	Self-administered	Topical, available over the counter.
Sinecatechin	Self-administered	Topical prescription required.
Tretinoin	Self-administered	Topical; requires a prescription.
Scalpel/forceps removal	By clinician	Manual extraction; painful and may not be suitable for children or those with many lesions; risk of spreading the infection if not performed correctly.

Source: https://www.aad.org/public/diseases/a-z/molluscum-contagiosum-treatment.

**Table 2:** Summary of efficacy and safety of Zelsuvmi versus existing therapies

Treatment	Mechanism of action	Efficacy	Safety profile	Application & convenience
Zelsuvmi (berdazimer gel 10.3%)	Topical nitric oxide release; antiviral and immunomodulatory action	~30% complete clearance at 12 weeks in RCTs^[7,8]^	Mild-moderate local reactions (erythema, pain); well-tolerated^[7,9]^	Self-applied, once daily; noninvasive^[7,10,11]^
Cantharidin	Vesicant; causes blistering and lesion detachment	Not suitable for all ages	Known for causing blisters, may need repeat application	Physician applied only; office-based^[6]^
Physical methods (e.g., curettage and cryotherapy)	Mechanical destruction of lesions	Generally, high clearance with a single treatment	Painful, risk of scarring; not well tolerated in children	Clinic-based, often requires anesthesia in pediatrics
Imiquimod	Immune response modifier (TLR7 agonist)	Shown to be ineffective in RCTs for MC^[6]^	Causes irritation, inflammation, poor tolerability^[6]^	Topical, but requires long duration of use
Cimetidine, KOH, tretinoin (off-label topicals)	Immune modulation or keratolytic action	Beneficial in atrophic dermatitis	Skin irritation common	Varies by agent; often used off-label
No treatment/watchful waiting	Natural immune clearance over time	Most cases resolve in 6–12 months^[4,6]^	No adverse effects	No intervention needed

## Zelsuvmi: a breakthrough treatment

The U.S. Food and Drug Administration (FDA) has sanctioned ZELSUVMI™ (10.3% berdazimer topical gel) for MC treatment in both adults and pediatric patients aged 1 year and above. This approval marks a significant milestone as ZELSUVMI becomes the first innovative drug to target molluscum infections^[10]^.

## Mechanism of action

Zelsuvmi, a topical gel containing 10.3% berdazimer, a nitric oxide (NO) releasing agent, operates through a unique mechanism of action, primarily targeting the MCV and its replication cycle. Berdazimer, the active ingredient, acts as a potent inhibitor of viral replication by disrupting key molecular pathways involved in viral propagation. Specifically, berdazimer interferes with viral DNA synthesis and assembly, ultimately leading to the inhibition of viral replication and spread. Additionally, berdazimer exhibits immunomodulatory properties, enhancing the body’s natural defense mechanisms against MCV infection. This dual-action mechanism not only suppresses viral proliferation but also promotes the resolution of existing lesions, offering patients a comprehensive treatment approach for MC^[11]^. It additionally uses cytotoxic reactive oxygen and nitrogen compounds to influence viral replication^[3]^.

## Key discoveries from the berdazimer trials

The study conducted by Browning *et al*, as part of the B-SIMPLE4 trial, serves as a compelling example, highlighting the pivotal significance of patient-centered outcomes in the assessment of treatment effectiveness in which patients treated with berdazimer experienced not only a significant reduction in visible lesions but also notable improvements in quality of life and emotional well-being. By week 12, 82% of participants receiving berdazimer reported substantial improvement in lesions compared to 60% in the vehicle group. This trend continued at week 24, with 84% of the berdazimer group and 71% of the vehicle group reporting ongoing improvement, underscoring berdazimer’s effectiveness as a treatment option^[12]^.

Another study by Tomoko *et al*, summarizing findings of B-SIMPLE2 and BSIMPLE1 trials, showed that patients presenting as BOTE-positive (BOTE – beginning of the end – describes inflammation such as redness, swelling, and scaling in the evolution of MC that signals the body’s immune response and often marks the near resolution of MC^[12]^) who underwent SB206 therapy exhibited the most pronounced reduction in MC lesion counts, reflecting that SB206 elicits BOTE markers and may expedite the resolution of MC infection. By week 12, MC lesion counts had diminished by 50.7% from baseline in BOTE-positive cases compared to a 29.1% reduction for BOTE-negative cases (*P* = 0.0015) within the vehicle-treated cohort. Conversely, for the SB206-treated cohort, baseline BOTE-positive patients experienced a 63.3% reduction, whereas BOTE-negative patients saw a 51.7% decrease (*P* = 0.0194). Local skin reactions, primarily application site pain and erythema, were the most frequently reported adverse effects^[13]^.

The cornerstone in establishing the safety and efficacy of Zelsuvmi in MC care is the B-SIMPLE4 trial, the largest randomized clinical trial investigating MS treatment to date. This Phase 3 study enrolled 891 participants aged ≥6 months across 55 dermatology and pediatrics clinics in the USA. Zelsuvmi exhibited statistically significant efficacy, with 32.4% of patients achieving complete resolution of all MC lesions at week 12, compared to only 19.7% in the vehicle group. This finding emphasizes a marked disparity in treatment outcomes between the two cohorts. Adverse events were predominantly tolerable, with application-site pain and erythema being the most frequently reported occurrences, which most likely represent an anticipated pharmacological effect of topical NO application. Notably, no systemic treatment-related adverse events, except for crying and insomnia, were reported^[8]^. A study by Cartwright *et al* demonstrated that berdazimer gel (10.3%) has favorable pharmacokinetics with minimal systemic exposure, supporting its safety profile^[9]^. Similarly, in a comprehensive study by Jeffrey *et al*, which combined data of all B-SIMPLE trials involving 1598 patients (917 on berdazimer and 681 on vehicle), berdazimer showed superior efficacy at week 12, with complete clearance rates of 30.0% compared to 19.8% in the vehicle group (OR: 1.75; 95% CI: 1.38–2.23, *P* < 0.001). Subgroup analyses confirmed consistent efficacy across various demographic and clinical profiles, including age, sex, lesion count, and disease duration^[7]^.

In patients with atopic dermatitis, a study by Paller *et al* demonstrated that at week 12, 35% of those treated with berdazimer achieved complete lesion clearance, compared to 27.4% in the vehicle group in the positive atopic dermatitis group. Adverse events were mostly mild, with skin irritation and application site erythema reported by 12% of AD-negative patients. Collectively, these findings support berdazimer gel as a promising, effective, and well-tolerated option for treating MC, even in patients with atopic dermatitis^[14]^ (trials summarized in Table 3).

**Table 3:** Critical comparison of Zelsuvmi with other emerging treatments

Study ID	Intervention	Phase	Sample size	Outcomes	Adverse events
NCT04535531 (B-SIMPLE4)	SB206 10.3% berdazimer: topically once daily versus vehicle gel: topically once daily	Phase 3	891 participants were randomized, 444 to berdazimer 10.3%, 51.4% male, and 447 to vehicle, 52.3% female.	The primary efficacy endpoint was complete clearance of all MC lesions at week 12 (32.4% in SB206 versus 19.7% in placebo).	Application-site pain (18.69%) and application-site erythema (11.71%) were the most frequent treatment-emergent adverse events (TEAEs), and most TEAEs were mild or moderate.
NCT03927703 (B-SIMPLE2)	Subjects or their caregivers will apply SB206 12% or vehicle gel once daily for a minimum of 4 weeks and up to 12 weeks to all lesions identified at baseline and new treatable lesions that arise during the course of the study.	Phase 3	355 subjects ≥6 months of age with MC randomized 2:1 (active: vehicle).	The complete clearance rates at week 12 were 30% versus 20.3% for ZELSUVMI and vehicle, respectively, with a treatment difference of 9.2%.	Local skin reactions, primarily application site pain and erythema, occurred in 15.61 and 10.97% of people receiving SB206, respectively. 1/237 (0.42%) receiving SB206 12% reported cellulitis.
NCT03927716 (B-SIMPLE1)	Subjects or their caregivers will apply SB206 12% or vehicle gel once daily for a minimum of 4 weeks and up to 12 weeks to all lesions identified at baseline and new treatable lesions that arise during the course of the study.	Phase 3	352 subjects ≥6 months of age with MC randomized 2:1 (active: vehicle).	The complete clearance rates at week 12 were 25.8% versus 21.6% for ZELSUVMI and vehicle, respectively, with a 95% confidence interval (−5%, 14%).	Local skin reactions, primarily application site pain and erythema, occurred in 21.7% and 12.34% of people receiving SB206, respectively. Application site scar was also reported in few cases.

Zelsuvmi’s potential to revolutionize MC management is evident in the aforementioned studies. Its superior efficacy, swift clearance times, and patient-friendly nature mark a fundamental shift in MC treatment, presenting improved outcomes for patients and healthcare system.

## Recommended dosage and adverse effects

There are two tubes included with ZELSUVMI. About 14 g of berdazimer gel is contained in Tube A, which has a blue label, and 17 g of hydrogel is included in Tube B, which has a yellow label. Prior to application, combine equal parts of the gel from Tubes A and B and apply evenly on the lesion, once daily for 12 weeks^[10]^. The majority of adverse reactions (≥1%) that are reported are application site reactions, which include dermatitis (4.9%), swelling (3.5%), erosion (1.6%), discoloration (1.5%), vesicles (1.5%), irritation (1.2%), pruritus (5.7%), erythema (11.7%), pain (such as burning or stinging sensations, 18.7%), exfoliation (5.0%), and infection (1.1%)^[10]^

## Healthcare professional perspectives

Zelsuvmi has been recognized for its potential therapeutic benefits. However, to maximize its efficacy and safety, it is essential to adhere to the best practices for its usage. First, Zelsuvmi should be used strictly as directed by healthcare professionals. The prescribed dosage and frequency of application should not be exceeded, as this can lead to adverse reactions or diminished effectiveness. The drug should be applied evenly across the affected area, avoiding contact with the eyes and mucous membranes to prevent irritation. Secondly, patients should be aware of the potential side effects of Zelsuvmi, which may include skin irritation, redness, and itching. In case of severe or persistent side effects, immediate medical attention should be sought and if these symptoms manifest, patients should cease using berdazimer and commence appropriate therapy to alleviate the symptoms of allergic contact dermatitis^[10,15]^. Thirdly, it is important to note that Zelsuvmi may interact with certain other medications^[16]^. Therefore, patients should consult their healthcare provider before using Zelsuvmi in conjunction with other drugs. Special caution should be exercised by pregnant or breastfeeding women, and individuals with specific health conditions, who should seek medical advice before using Zelsuvmi^[16]^.

## Future directions

The FDA approval of Zelsuvmi represents a pivotal advancement in dermatological care, particularly in managing MC. This approval paves the way for healthcare providers to access and prescribe Zelsuvmi, expanding the armamentarium against MC. Its FDA approval addresses a significant medical need, giving healthcare providers and patients a reliable and effective treatment option. This progress not only improves patient outcomes but also enhances the quality of life for those affected by this condition. In conclusion, the best practices for Zelsuvmi usage involve strict adherence to the prescribed regimen, awareness of potential side effects, and consultation with healthcare professionals in case of doubt or adverse reactions. To further understand its long-term advantages and maximize its application in clinical practice, further study and clinical experience will be necessary as we proceed. By opening the door to better results and a higher standard of living, Berdazimer’s clearance gives both patients and doctors fresh hope.

## Conclusion

MC infection presents as small, raised bumps and spreads through direct skin contact and contaminated objects. Zelsuvmi, with its innovative NO therapy using berdazimer, offers a promising new treatment by directly targeting the virus’s replication cycle and boosting the body’s immune response.

## Data Availability

Not available.

## Research registration unique identifying number (UIN)

Not applicable, as this is a review article.

## Permission to reproduce material from other sources

This study has not used any material that needs prior permission.
